# Representation of female authors in oncology: the Indian perspective

**DOI:** 10.3332/ecancer.2024.1755

**Published:** 2024-09-05

**Authors:** Vanita Noronha, Manali Kolkur, Chinmay Haridas, Priyanka Bhagyavant, Richa Das, Shrusti Sagaraih Chittari, Lakshanya Vasudevan, Gunj Bafna, Nandini Menon, Minit Shah, Kumar Prabhash

**Affiliations:** 1 Medical Oncology, Tata Memorial Hospital, Homi Bhabha National Institute, Mumbai 400012, India; 2 Department of Medicine, Shri Vasantrao Naik Government Medical College, Yavatmal 445001, India; 3 Sunrise Oncology Center, Mumbai 400092, India; a https://orcid.org/0000-0001-8858-5004

**Keywords:** female authorship, oncology, high-impact journals, first author, gender

## Abstract

**Background:**

Despite an increasing number of female oncologists, disparities persist in authorship representation of women, especially in high-impact journals.

**Objective:**

This study aimed to investigate gender differences in authorship within select high-impact Indian oncology journals over a 5-year period, assessing trends in the gender gap.

**Methods:**

Six high-impact Indian oncology journals were selected for analysis. Data on original articles, reviews and editorials published between 2017 and 2022 were collected, including authors’ gender, their role as first author or corresponding author and name of the journal. Gender determination was validated through web searches. Descriptive statistics were used to summarize the data and to study the prevalence of female authorship across journals.

**Results:**

A total of 2,235 articles were included. Across all journals, 30.4% of authors were female, with Journal of Cancer Research and Treatment exhibiting the highest proportion of female authors (948/2,507; 37.8%). Female authorship increased over time, with first authors rising from 33% to 41%, and corresponding authors from 29.4% to 36.4%. However, disparities persisted, and certain journals exhibited fluctuating trends. Female authorship was higher in original articles (30.9%) compared to reviews (27.8%) and editorials (24.5%). Women comprised 3.5%–24.4% of the editorial boards of the six journals.

**Conclusion:**

Female representation, both as authors and editorial board members of Indian oncology journals is disproportionately low. Proactive measures are necessary to address these disparities and promote gender equity in academic publishing.

## Introduction

The advancement of the healthcare profession is heavily influenced by scientific contributions, but research in this domain highlights a noticeable disparity in the gender representation among authors involved in producing scientific content. Ross *et al* [1] reported that there was a 13.2% gender gap for articles and a 58.4% gap for patents. They found that this gender gap in authorship was, at least partly, due to a lack of attribution or credit, i.e., although both men and women had done the research work, women were less likely to be given authorship [1].

Research productivity is widely considered an essential benchmark for professional advancement, leading to increased opportunities for tenure and leadership positions [2]. However, several studies have reported staggeringly low numbers of female authors in journals of various disciplines of medicine, including oncology [3, 4]. Jagsi *et al* [3] studied the articles published by 7,249 American physicians and reported that the proportion of female first authors increased from 5.9% in 1970 to 29.3% in 2003. There was a similar increase in the proportion of female corresponding authors, which increased from 3.7% in 1970 to 19.3% in 2003. The proportion of female authorship was the lowest in the Annals of Surgery, while the increase in female authorship was substantial in two other journals: Obstetrics and Gynaecology, and the Journal of Paediatrics [3]. In an analysis of 420,526 authors from 58,368 oncology articles (from the three disciplines in oncology), Yalamanchali *et al* [5] reported that 29.5% of the authors were female. Hart and Perlis [4] reported that female authors in oncology increased from 37.6% in 2008 to 41.1% in 2017. Thus, although there has been a recent rise in female authorship, at least in some medical specialties such as Obstetrics/Gynaecology and Paediatrics but not in other traditionally male-dominated specialties like surgery, these numbers have not been commensurate with the rise in number of female medical faculty. In the USA, there was an approximately 9%–10% increase in female oncology faculty between 2002 and 2018, and almost 45% of the haematology–oncology trainees were women, yet the increase in female authorship of oncology articles was only 5.6% [4, 5].

In a report published in 2022, 35.8% of oncologists in the USA were women; unfortunately, these figures are not available for India [6]. In a survey done to examine the gender climate in Indian oncology institutions, Bajpai *et al* [7] reported that only 15.4% of the oncology professionals surveyed worked in teams with a majority of women, and only 32.7% had a woman manager. An editorial published by Chopra *et al* [8] reported that approximately half of the oncology trainees in India were women, yet only 17% of practicing Indian physicians were women. Additionally, there exists a dearth of literature on female authorship in high-impact Indian oncology journals over the past several years. We, therefore, investigated the gender differences in authorship and the editorial board composition of five high-impact Indian oncology journals, and examined the changes over time as well as the differences between the journals studied.

### Objective

The purpose of this study was to examine gender differences in authorship of manuscripts in select high‐impact Indian oncology journals, and to assess for any trends in the gender gap over time. We also aimed to study the gender composition of the editorial boards of these select Indian oncology journals.

## Methods

We selected six Indian journals with a high impact in the category ‘Oncology’ for the article types: original article, review and editorial. The six journals were South Asian Journal of Cancer, Indian Journal of Medical and Paediatric Oncology, Indian Journal of Cancer, Cancer Research, Statistics and Treatment, Journal of Cancer Research and Therapeutics and Indian Journal of Surgical Oncology. South Asian Journal of Cancer; Indian Journal of Medical and Paediatric Oncology; Indian Journal of Cancer and Cancer Research, Statistics and Treatment are run by medical oncology teams, and their focus is on medical oncology topics, although all varieties of oncology manuscripts are published. Journal of Cancer Research and Therapeutics is focussed on radiation oncology, while Indian Journal of Surgical Oncology is predominantly a surgical oncology journal.

For every journal, we listed all the original articles, review articles and editorials published between 2017 and 2022. Cancer Research, Statistics and Treatment first started publication in 2018, hence for this journal, we collected data from 2018 to 2022. We recorded the name of the journal, date of publication (year and month), gender of each author (female/male), total number of authors and total number of female authors. The authors of each retrieved article were classified according to their position in the list of authors, specifically as the first author, and/or the corresponding author. If there was more than one corresponding author, we considered the last/senior author to be the corresponding author for the purpose of our analysis. All the obtained data were tabulated to show the names of the authors if they were female, as well as the number of female authors for every article. The gender determination was done by two of our authors (KP and VN) and verified with results from a web search done using LinkedIn and ResearchGate. Gender-ambiguous names from Chinese and Turkish publications were excluded from the analysis.

### Statistical analysis

The data were collected and analysed in Microsoft Excel. Descriptive statistics were used to summarise the data and to calculate the proportion of female authorship in the selected journals. All categorical data were presented as numbers (percentages). To describe the time-trend of authorship, we calculated the equation of the line (slope and intercept). The *R*-squared value (*R*^2) in the trend line represents the proportion of the variance in the dependent variable (percentages of male and female authors) that is explained by the independent variable (year), indicating the goodness of fit of the linear regression model. Graphs were plotted with the help of Microsoft Excel.

## Results

We evaluated 2,235 articles published by 13,068 authors between 2017 and 2022, which comprised 1,823 (81.6%) original articles, 307 (13.7%) review articles and 105 (4.7%) editorials. Across all the journals, 30.4% of authors (3,976 of 13,068) were female and 69.6% (*n* = 9,092) were male. Female representation in the first author position was slightly higher at 34.0% (759 of 2,239) than in the position of the corresponding author at 30.5% (681 of 2,231). Female author representation in original articles was 30.9% (3,504 of 1,1343); that for review articles was 27.8% (419 of 1,509) and for editorials was 24.5% (53 of 216). Table 1 provides the details of the articles we reviewed from the six selected journals, and the percentages of female and male authors in each journal. Table 2 shows the proportion of female authors, in the published articles between 2017 and 2022. Table 3 provides the details of female representation as first authors and corresponding authors, both overall, and for each article type including original articles, review articles and editorials for the six journals.

**Figure 1.**
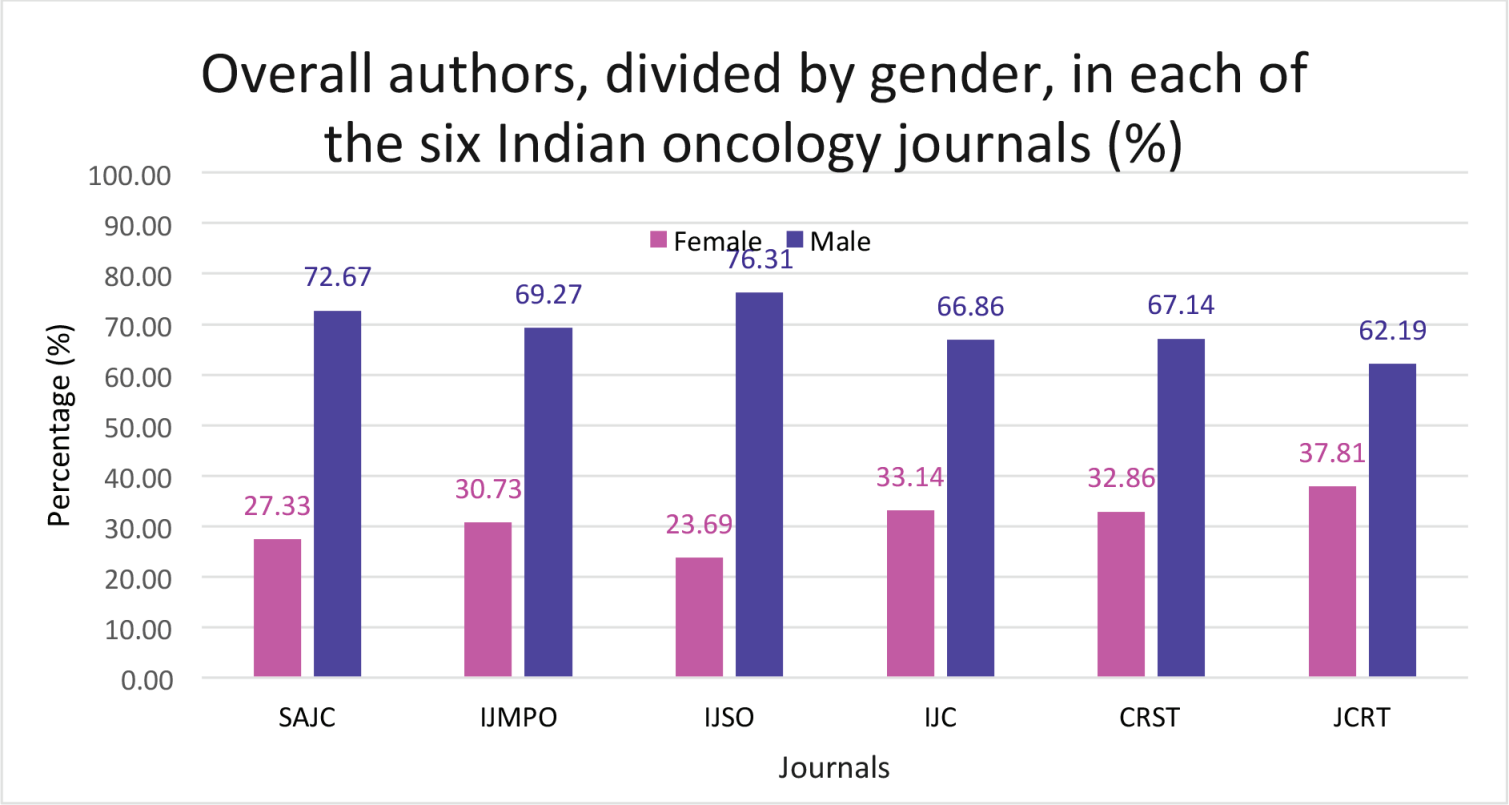
Total male versus female authors in each of the six Indian oncology journals (The numbers depicted are the percentage based on the total number of authors in that journal in the timeframe of the study). SAJC-South Asian Journal of Cancer, IJMPO-Indian Journal of Medical and Paediatric Oncology, IJC-Indian Journal of Cancer, CRST-Cancer Research, Statistics, and Treatment, JCRT-Journal of Cancer Research and Therapeutics, IJSO-Indian Journal of Surgical Oncology.

**Figure 2.**
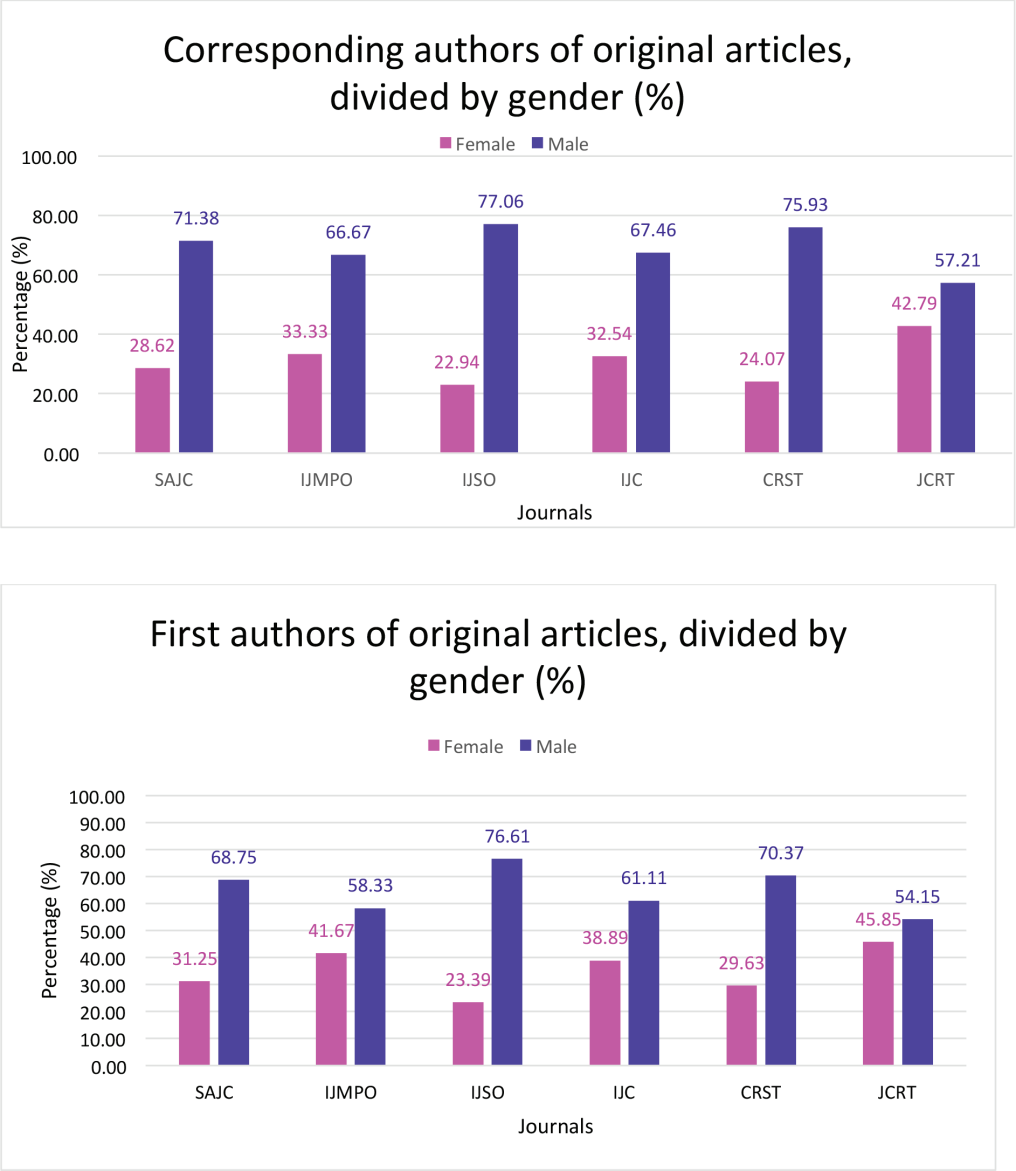
(a): Total male versus female corresponding authors in original articles across the six journals depicted by percentage. (b): Total male versus female as first authors in original articles across the six Indian oncology journals depicted by percentage. SAJC-South Asian Journal of Cancer, IJMPO-Indian Journal of Medical and Paediatric Oncology, IJC-Indian Journal of Cancer, CRST-Cancer Research, Statistics, and Treatment, JCRT-Journal of Cancer Research and Therapeutics, IJSO-Indian Journal of Surgical Oncology.

**Figure 3.**
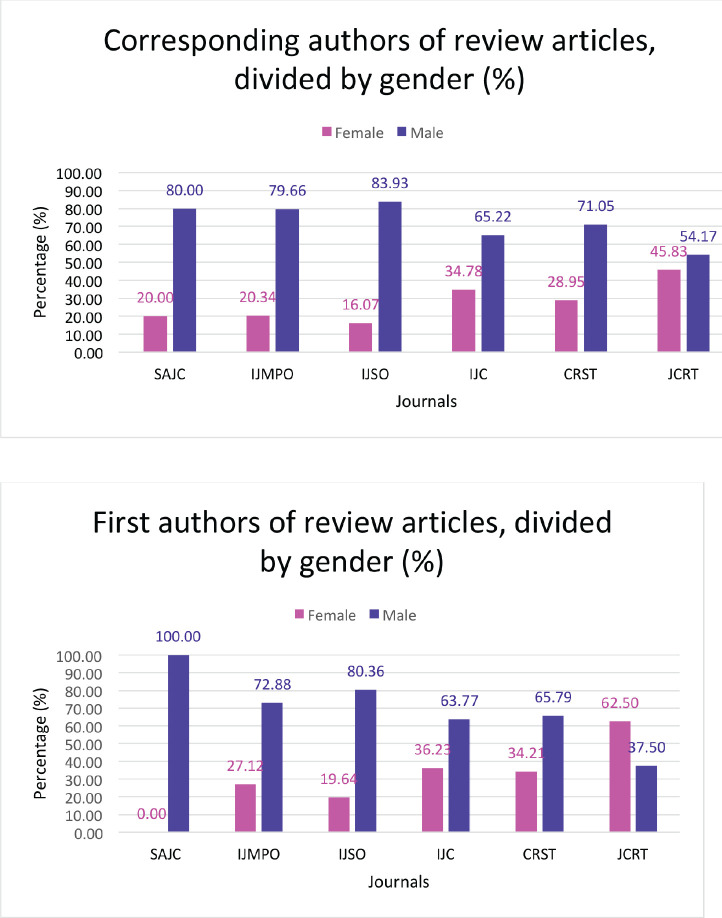
(a): Total male versus female as corresponding authors in review articles across the six Indian oncology journals depicted by percentage. (b): Total male versus female as first authors in review articles across the six Indian oncology journals depicted by percentage. SAJC-South Asian Journal of Cancer, IJMPO-Indian Journal of Medical and Paediatric Oncology, IJC-Indian Journal of Cancer, CRST-Cancer Research, Statistics, and Treatment, JCRT-Journal of Cancer Research and Therapeutics, IJSO-Indian Journal of Surgical Oncology.

Overall, the lowest representation of female authors was in the Indian Journal of Surgical Oncology at 23.7%, while the highest female author representation was in the Journal of Cancer Research and Therapeutics at 37.8% (Figure 1). There was a similar observation across the journals regarding female representation, both as first authors and as corresponding authors, with the Journal of Cancer Research and Therapeutics having the highest proportion of female first authors as well as corresponding authors at 46.4% and 42.7%, respectively; and the Indian Journal of Surgical Oncology having the lowest (22.8% and 21.8%, respectively). With respect to original articles, the Journal of Cancer Research and Therapeutics had the highest percentage of female authors (both first author (45.9%) and corresponding author (42.8%)), while the lowest was seen in the Indian Journal of Surgical Oncology (23.4% and 22.9%, respectively) (Figure 2a and b). Additionally, the Journal of Cancer Research and Therapeutics had the highest number of review articles with female first authors (62.5%) and corresponding authors (45.8%). The South Asian Journal of Cancer had the lowest number of female first-authored review articles (0), across all the selected years (Figure 3a and b).

**Figure 4.**
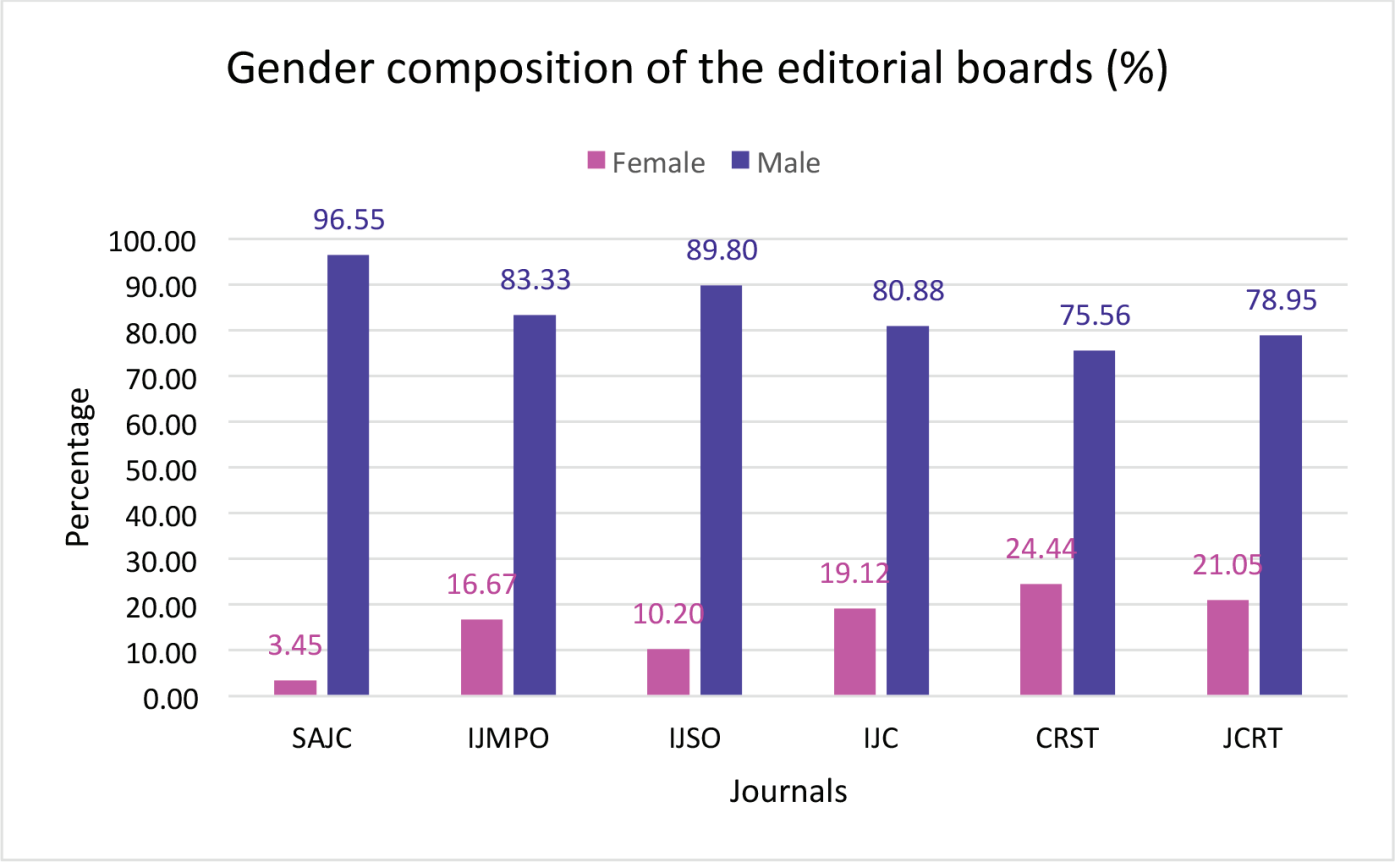
Total male versus female as members of the editorial board across the six Indian oncology journals depicted by percentage. SAJC-South Asian Journal of Cancer, IJMPO-Indian Journal of Medical and Paediatric Oncology, IJC-Indian Journal of Cancer, CRST-Cancer Research, Statistics, and Treatment, JCRT-Journal of Cancer Research and Therapeutics, IJSO-Indian Journal of Surgical Oncology.

On analysing the composition of the editorial boards across the six journals we found that Cancer Research, Statistics and Treatment was the only journal with a female editor-in-chief; coincidentally, this journal also had the highest proportion (11 of 45, 24.4%) of women on the editorial board. The South Asian Journal of Cancer had the lowest proportion of female representation on the editorial board (1 of 29, 3.5%) other journals also had low female editorial board representation: 10.2% in the Indian Journal of Surgical Oncology, 16.7% in the Indian Journal of Medical and Paediatric Oncology, 19.1% in the Indian Journal of Cancer and 21.1% in the Journal of Cancer Research and Therapeutics (Figure 4).

**Figure 5.**
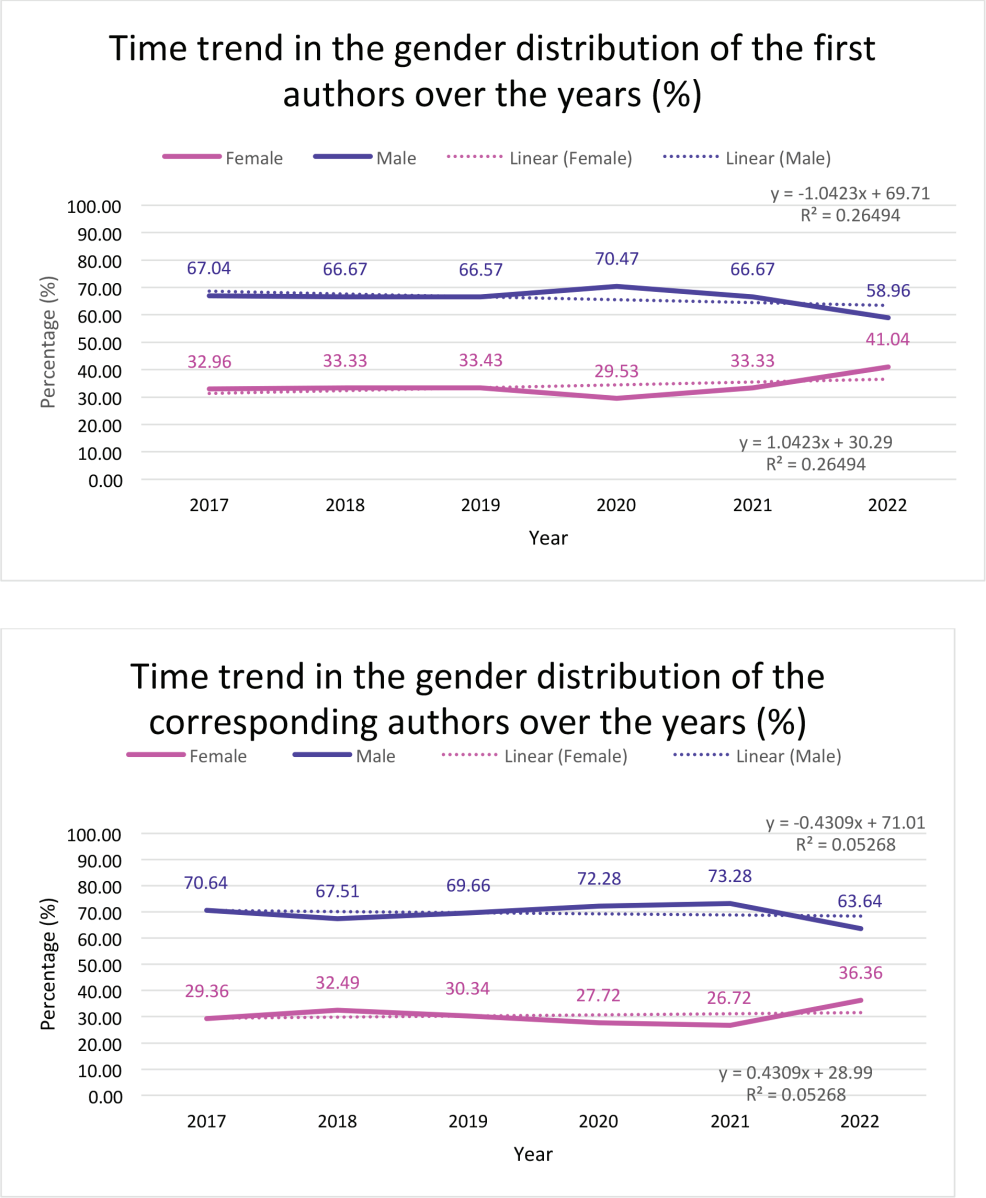
(a): Total male versus female as first authors through the years depicted by percentage. (b): Total male versus female as corresponding authors through the years depicted by percentage.

Over the 5 years from 2017 to 2022, the percentage of female first authors increased from 33% to 41% (*R*^2^ = 44.14%; Figure 5a). A similar increase was seen in the percentage of female corresponding authors (29.4% to 36.4%; *R*^2 = 83.83%) (Figure 5b).

## Discussion

In our study, we examined the gender gap in the authorship of six leading Indian oncology journals. We searched for original articles, review articles and editorials published between 2017 and 2022 and saw an increase in the percentage of female authors over time in the six selected journals. However, the gender gap persisted, which is in line with the data previously reported [9, 10]. Additionally, we found that female authorship was higher in original articles as compared to review articles and editorials across all the journals.

As of 2024, 48.44% of India’s population is female, compared to 51.56% male population [11]. In 2021–2022, women outnumbered men in medical schools (57.6% versus 42.4%) [12]. However, only about a third of positions at the postgraduate and doctoral level were filled by women [13]. When it comes to practice, the numbers are dismal. As per a report from the World Health Organisation, only 14.2% of the medical doctors in India in 2004 were women; 85.8% were men [14]. In our study, we found that 30.7% of the authors of articles in Indian oncology journals were female and more than double (69.3%) the authors were male. This is in line with data from several countries showing the percentage of female authors to be significantly lower than that of male authors, although this percentage increased during the course of those respective studies [15, 16]. Studies from the Indian sub-continent also echoed our findings, including one study that analysed gender disparities in Indian journals [16–18]. Bajpai *et al* [7] assessed 558 articles published in the Indian Journal of Cancer and the Indian Journal of Medical and Paediatric Oncology between 2017 and 2018. They reported that 26% of these articles had women as first or corresponding authors; this finding was similar to our study [7]. This gender disparity in Indian oncology publications is reflective of the broader picture of gender inequality in the country. According to the United Nations Human Development Report 2023–2024, India ranks 108th (of a total of 193 countries) in the gender inequality index (a composite measure reflecting inequality between females and males in reproductive health, empowerment and the labour workforce). In India, as of 2022, although 41% women and 58.7% men had at least some secondary school education, only 28.3% of women were a part of the workforce, compared to 76.1% of the men, and only 14.6% of the parliament seats were held by women [19]. Addressing this gender inequality on a broad scale in India will likely lead to the shrinking of the gender gap in multiple areas, including in oncology.

Hearteningly, we saw an overall increase in the percentage of female authorship in the six selected journals from 2017 to 2022. This increase, however, was not a linear one and was not spread evenly across the journals. In three journals (Indian Journal of Surgical Oncology, South Asian Journal of Cancer and Indian Journal of Cancer), female author representation decreased from 2019 to 2020, and then again increased in 2021. We were not able to assess the reasons for the increase/decrease for each journal specifically. However, issues that have been previously reported include a lesser percentage of female department leaders and full professors in academic institutions [9], female investigators receiving less intramural and extramural grants [20], and that the gender of the editor-in-chief may influence article submissions and priorities [21].

We found that there was a higher representation of women as first authors (34.2%) than as corresponding authors (30.5%). This was similar to the findings from studies done globally. Hart and Perlis [4] found that in global oncology publications in 2017, 46.6% had women first authors, whereas only 32.7% had women senior authors. They reported that articles in which women were the last/senior author had a 13% higher likelihood of having a female first author, as compared to articles with a male senior author, *p* < 0.001. They also noted that women required approximately double the time (10 years) to transition to the last author position as compared to men (5 years) [4]. Similar findings were reported by Yalamanchali *et al* [5] from publications in 13 general oncology/medicine, radiation oncology and surgery journals between 2002 and 2018. They found that it was significantly less likely for a woman to occupy the last author position than the first author position; odds ratio, 0.60; 95% CI, 0.52–0.69 [5]. Hornstein* et al* [9] studied 5,302 authors who had published 608 articles in the Journal of Clinical Oncology Global Oncology between October 2015 and March 2020. They also found that women were most under-represented as last authors (32.1%), as opposed to first authors (41.4%) or middle authors (38.1%) [9]. In publications from low- and middle-income countries, women were the last authors in 18.9% cases, and the proportion was the lowest for manuscripts from South Asia at 14.5%. Bajpai *et al* [7], who had analysed the authorship details of publications in two Indian oncology journals over 2 years (2017–2018) had clubbed first and lead authorship positions together to study the overall female representation; hence, we were unable to compare our results with theirs [7].

We found that female authorship was higher in original articles (30.9%) as compared to review articles (27.8%) and editorials (24.5%) across all the journals. This is similar to what was reported by Jagsi *et al* [3] who found that in 2004, only 11.4% of the authors of guest editorials in the New England Journal of Medicine and 18.8% in the Journal of the American Medical Association (JAMA) were female; compared to 29.3% overall female first authors for original research articles. Contrarily, Hornstein *et al* [9] reported that 60.5% of review articles had women first authors versus 32.6% male; approximately 38% of the first authors of editorials were female, although there were only 16 editorials included in their analysis. A possible explanation for the lower representation of women in review articles and editorials is that these are often commissioned by the journal. Given the heavily male-dominated composition of the editorial boards of all the six journals that were included in our study, it is possible that this may have led to invitations to a larger number of male colleagues due to implicit gender bias. Another possible reason proposed by Jagsi *et al* [3] was the limited number of senior women faculty members who could contribute to review articles and editorials and the limited time of these senior women faculty, given the competing responsibilities of children and family. Riaz *et al* [22] had found that in American academic haematology-oncology programs, the proportion of women became smaller as the academic rank increased: 45.4% of Assistant Professors, 35.7% of Associate Professors and only 21.9% of full Professors were women. A mere 16.7% of Division Chiefs were women [22].

We found a stark gender inequality in the editorial boards of the journals we selected. The highest proportion of women members of the editorial board was 24.4% for the Cancer Research, Statistics and Treatment. This was also the only journal of the six (16.7%) that had a female editor-in-chief. Women comprised between 3.5% and 24.4% of the editorial boards of the six journals. It is possible that the male dominance of the editorial board contributed at least partly to the gender inequality in the articles selected for publication in the journals. A recent editorial reported a far better gender distribution in the JAMA Network of journals. In a recent survey completed in January 2024, it was found that women represented 44% of the editors and board members of JAMA and its affiliate journals, which had increased by 6% from when the same survey was earlier conducted in 2021. Five of the 13 editors-in-chief (38.5%) were women [21].

In terms of the gender composition of the articles in the individual journals, the Indian Journal of Surgical Oncology had the poorest female representation while the Journal of Cancer Research and Therapeutics (radiation oncology journal) had the highest female representation, both for first and corresponding authors. This is likely reflective of the gender distribution among the faculty in radiation and surgical oncology in India. An article by Sarkar and Pradhan [23] reported that 34.1% of the radiation oncologists in India are women, compared to approximately 26% in the USA. Surgery has traditionally been a male-dominated field, and only 22% of American surgeons and 12.5% of Indian surgeons are women [24]. Jagsi *et al* [3] had also reported that the Annals of Surgery had the lowest proportion of women senior authors (6.7%) in 2004. Whether the dismal female representation in surgical oncology articles is simply a result of low numbers of female surgical oncology faculty or is also reflective of bias and/or gender discrimination is uncertain. Pandrowala *et al* [25] had reported that in their surgical oncology department, 28% of female trainees (compared to 6.6% of male trainees) and 26.6% of female faculty (compared to 14.8% of male faculty) reported facing gender discrimination.

There were certain limitations to this work. Our analysis did not include oncology publications by Indian authors in international oncology journals, or in non-oncology journals. We did not include transgender as a part of this analysis. Furthermore, we had put a mechanism in place to identify the gender of the authors but were unable to confirm individually with all the authors. It is possible that the gender skew in the publications may have been contributed by** the volume of articles submitted to each journal or because of a higher rejection rate. We were unable to access the details of the rejected articles and were only able to analyse the articles that had been published. Analysing the reasons for the gender gap was beyond the scope of our study. However,** our study is the first organized effort to provide detailed data on female participation in Indian oncology publications. This also provides data for female contributions as decision makers in the editorial boards of Indian oncology journals. We hope that this initial analysis will provide an impetus towards more gender equity in Indian oncology publications. Some immediately actionable solutions would be to make the editorial boards more gender balanced. An unblinded peer review process may introduce bias towards articles written by male authors, and therefore, a double-blinded peer review process should be encouraged. In the broader context, political reform, particularly in academic programmes, to make the work place more woman-friendly, would go a long way towards ensuring a more gender-neutral oncology workforce.

## Conclusion

Less than a third of the articles in the top six oncology journals of India are authored by women. Female representation in the editorial boards of these journals ranges from 4% to 24%, and only one of the journals has a female editor-in-chief. There is an urgent need to take proactive measures to improve this situation.

## Conflicts of interest

No author has conflict of interest.

## Funding

None.

## Ethics approval

Not applicable.

## Patient consent

Not applicable.

## Permission to reproduce material from other sources

Not applicable.

## Data availability

The individual de-identified data will be available on request to the corresponding author, Dr Kumar Prabhash (kumarprabhashtmh@gmail.com), until 5 years after publication. Requests beyond this timeframe will be considered on a case-by-case basis.

## Author contributions

VN and KP were responsible for the conception and design of the study. All authors were responsible for curation of the data. VN, KP, PB and MK were responsible for analysis of the data. All authors were responsible for interpretation of the data. VN and CH drafted the article, and all other authors revised it critically for important intellectual content.

## Figures and Tables

**Table 1.** Overall gender distribution of authors across six Indian oncology journals.

Name of the journal	Number of articles reviewed (%) *n* = 2,235	Authors divided by gender, in number (%)		
		Female *n* = 3,976 (30.4%)	Male *n* = 9,092 (69.6%)	Total *n* = 13,068
South Asian Journal of Cancer	314 (14.0)	609 (27.3)	1,619 (72.7)	2,228
Indian Journal of Medical and Paediatric Oncology	326 (14.6)	575 (30.7)	1,296 (69.3)	1,871
Indian Journal of Surgical Oncology	568 (25.4)	738 (23.7)	2,377 (76.3)	3,115
Indian Journal of Cancer	342 (15.3)	737 (33.1)	1,487 (66.9)	2,224
Cancer Research, Statistics and Treatment	200 (8.9)	369 (32.9)	754 (67.1)	1123
Journal of Cancer Research and Therapeutics	485 (21.7)	948 (37.8)	1,559 (62.2)	2,507

**Table 2.** Female author representation for each year from 2017 to 2022 in the six Indian oncology journals.

	Number (%) of authors, divided according to gender											
Year of publication of the article	South Asian Journal of Cancer (*n* = 2,228)		Cancer Research, Statistics and Treatment (*n* = 1,123)		Journal of Cancer Research and Therapeutics (*n* = 2,507)		Indian Journal of Surgical Oncology (*n* = 3,115)		Indian Journal of Medical and Paediatric Oncology (*n* = 1,871)		Indian Journal of Cancer (*n* = 2,224)	
	Female	Male	Female	Male	Female	Male	Female	Male	Female	Male	Female	Male
2017	66 (23.07)	220 (76.92)	-	-	115 (38.72)	182 (61.27)	70 (20.83)	266 (79.16)	106 (34.08)	205 (65.91)	179 (23.39)	586 (76.60)
2018	118 (24.58)	362 (75.41)	14 (18.66)	61 (81.33)	127 (36.70)	219 (63.29)	97 (20.77)	370 (79.22)	95 (34.93)	286 (75.06)	170 (37.61)	282 (62.38)
2019	115 (30.66)	260 (69.33)	35 (29.91)	82 (70.08)	133 (31.97)	283 (68.02)	105 (25.42)	308 (74.57)	136 (31.05)	302 (68.94)	100 (39.52)	153 (60.47)
2020	90 (26.94)	244 (73.05)	126 (34.23)	242 (65.76)	167 (36.70)	288 (63.29)	84 (17.94)	384 (82.05)	83 (31.55)	180 (68.44)	95 (27.94)	245 (72.05)
2021	98 (29.51)	234 (70.48)	97 (31.29)	213 (68.70)	178 (36.70)	307 (63.29)	215 (26.54)	595 (73.45)	52 (21.84)	186 (78.15)	71 (48.63)	75 (51.36)
2022	122 (28.97)	299 (71.02)	97 (38.33)	156 (61.66)	228 (44.88)	280 (55.11)	167 (26.89)	454 (73.10)	103 (42.91)	137 (57.08)	122 (45.52)	146 (54.47)

**Table 3.** Female representation, for first and corresponding author, both overall, and for various article types including original articles, review articles, and editorials across six selected Indian oncology journals.

Name of the journal	First author				Corresponding author			
	Overall	Original articles	Review articles	Editorials	Overall	Original articles	Review articles	Editorials
South Asian Journal of Cancer	95 of 314 (30.2)	95 of 304 (31.2)	0 of 5 (0)	0 of 5 (0)	88 of 314 (28.0)	87 of 304 (28.6)	1 of 5 (20)	0 of 5 (0)
Indian Journal of Medical and Paediatric Oncology	123 of 326 (37.7)	105 of 252 (41.7)	16 of 59 (27.1)	2 of 15 (13.3)	98 of 326 (30.1)	84 of 252 (33.3)	12 of 59 (20.3)	2 of 15 (13.3)
Indian Journal of Surgical Oncology	130 of 568 (22.8)	105 of 449 (23.4)	22 of 112 (19.6)	3 of 7 (42.9)	124 of 568 (21.8)	103 of 449 (22.9)	18 of 112 (16.1)	3 of 7 (42.9)
Indian Journal of Cancer	128 of 342 (37.4)	98 of 252 (38.9)	25 of 69 (36.2)	5 of 21 (23.8)	114 of 342 (33.3)	82 of 252 (32.5)	24 of 69 (34.8)	8 of 21 (38.1)
Cancer Research, Statistics and Treatment	58 of 200 (29.0)	32 of 108 (29.6)	13 of 38 (34.2)	13 of 54 (24.1)	50 of 200 (25.0)	26 of 108 (24.1)	11 of 38 (28.9)	13 of 54 (24.1)
Journal of Cancer Research and Therapeutics	225 of 485 (46.4)	210 of 458 (45.8)	15 of 24 (62.5)	0 of 3 (0)	207 of 485 (42.7)	196 of 458 (42.8)	11 of 24 (45.8)	0 of 3 (0)
Total	759 of 2,235 (34.0)	645 of 1,823 (35.4)	91 of 307 (29.6)	23 of 105 (21.9)	681 of 2,235 (30.5)	578 of 1,823 (31.7)	77 of 307 (25.1)	26 of 105 (24.7)
